# Supplementary material for: The Roles of ROS Generation in RANKL-Induced Osteoclastogenesis: Suppressive Effects of Febuxostat
Source: Cancers (Basel). 2020 Apr 9;12(4):929. doi: 10.3390/cancers12040929 (PMC7226249; doi:10.3390/cancers12040929)
Supplement: Supplementary file 1 [file cancers-12-00929-s001.pdf]

Supplementary materials:

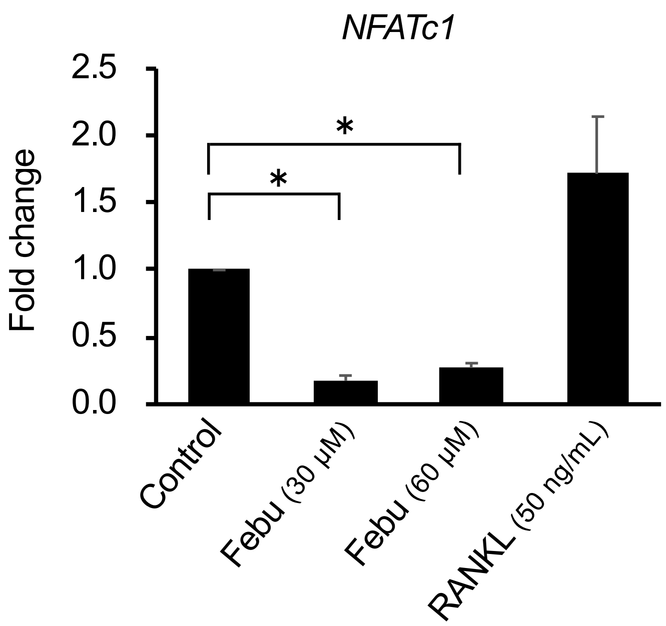

Figure S1. RAW264.7 cells were treated with indicated reagents for 24 hours. The gene expression of *NFATc1* was detected by real-time PCR. The results are expressed using delta-delta Ct method. *GAPDH* was used as an endogenous control to normalize each sample. Data were expressed as mean ± SD. *p <0.05.

Supplementary Figure 1

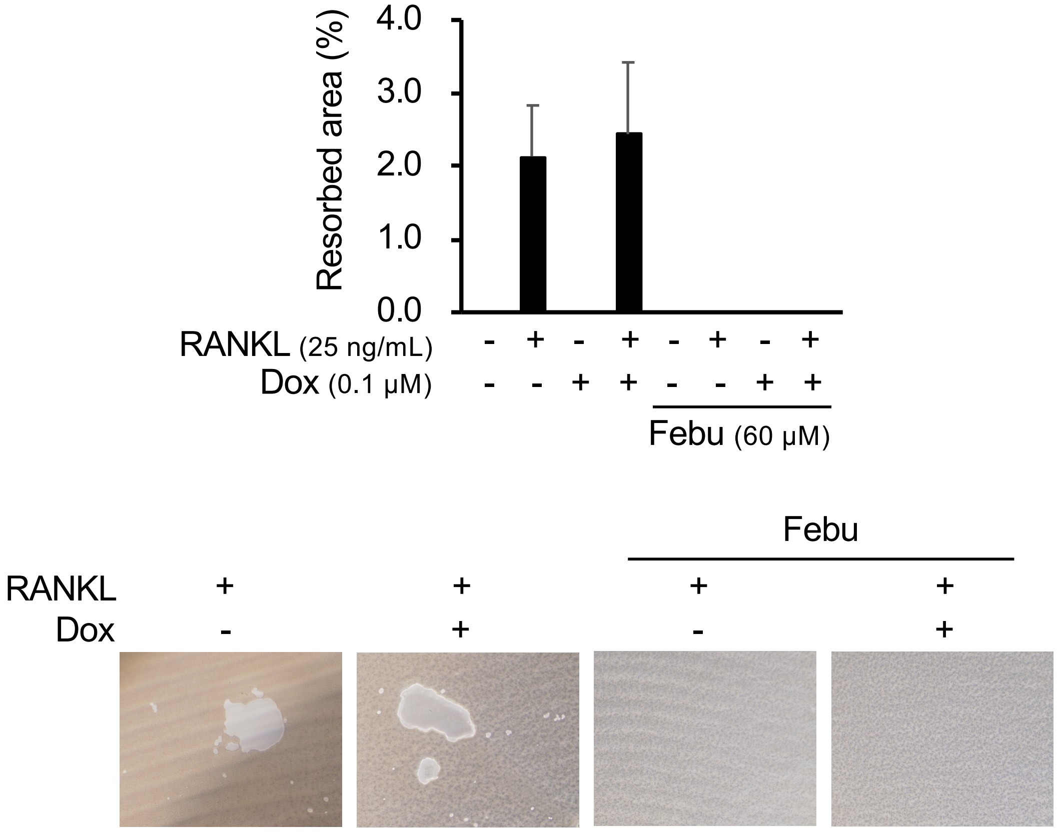

Figure S2. Bone marrow macrophages (BMMs) were cultured on 10 cm dish with M-CSF (10 ng/mL) and RANKL (50 ng/mL) for 7 days to generate mature OCs. The mature OCs were re-suspended in 15 mL tube after trypsinization and seeded onto Osteo-Assay Surface 96-well plates and cultured in triplicate with indicated reagents and M-CSF (10 ng/mL) for 2 days. Data are expressed as mean ± SE.

Supplementary Figure 2

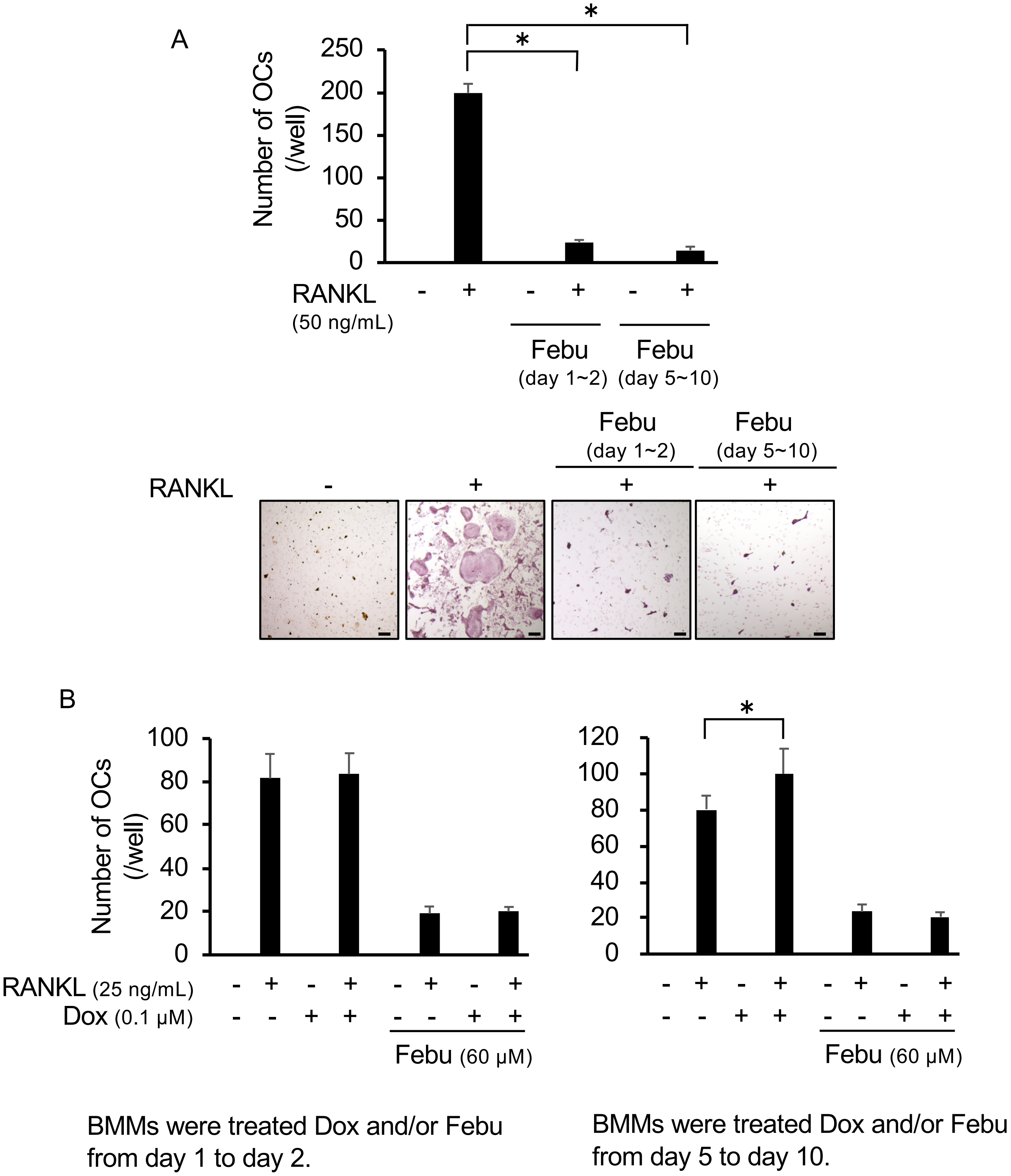

Figure S3. (A) Bone marrow macrophages (BMMs) were cultured in quadruplicates with M-CSF (10 ng/mL) and RANKL (50 ng/mL) for 10 days. Cells were treated with Febuxostat (Febu) at 60 μM from day 1 to 2 or from day 5 to 10. TRAP-positive cells containing three or more nuclei per well were counted. Data are expressed as mean ± SD. *p <0.05. Representative photos are shown. Original magnification, ×100. Bar, 100 μm. **(B)** BMMs were cultured in triplicates with M-CSF (10 ng/mL) and RANKL (25 ng/mL) in the presence or absence of Dox (0.1 μM) or Febu (60 μM) for the initial 2 days. Then, the culture medium was changed and the cells were cultured M-CSF (10 ng/mL) and RANKL (25 ng/mL) for the additional 8 days (left). BMMs were cultured in triplicates with M-CSF (10 ng/mL) and RANKL (25 ng/mL). At day 5, Dox (0.1 μM) and/or Febu (60 μM) was added and cultured for 5 days. TRAP-positive cells containing three or more nuclei per well were counted at day 10. Data are expressed as mean ± SD. *p <0.05.

Supplementary Figure 3

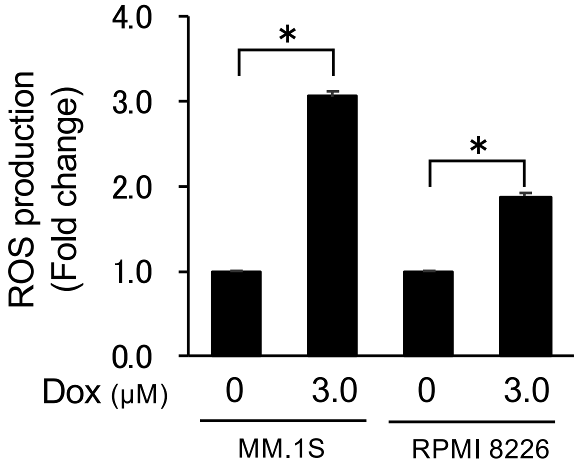

Figure S4. MM.1S and RPMI 8226 cells were pre-treated with H2DCFDA reagent at 10 μM for 30 minute. Cells were washed and re-suspended in culture media, and then the cells were treated with Dox (3.0 μM) for 2 hours. Cells were washed and re-suspended in PBS. ROS production in MM cells was detected with microplate reader SpectraMax i3 (Molecular Devices, California, USA). Data are expressed as mean ± SD. *p <0.05.

Supplementary Figure 4

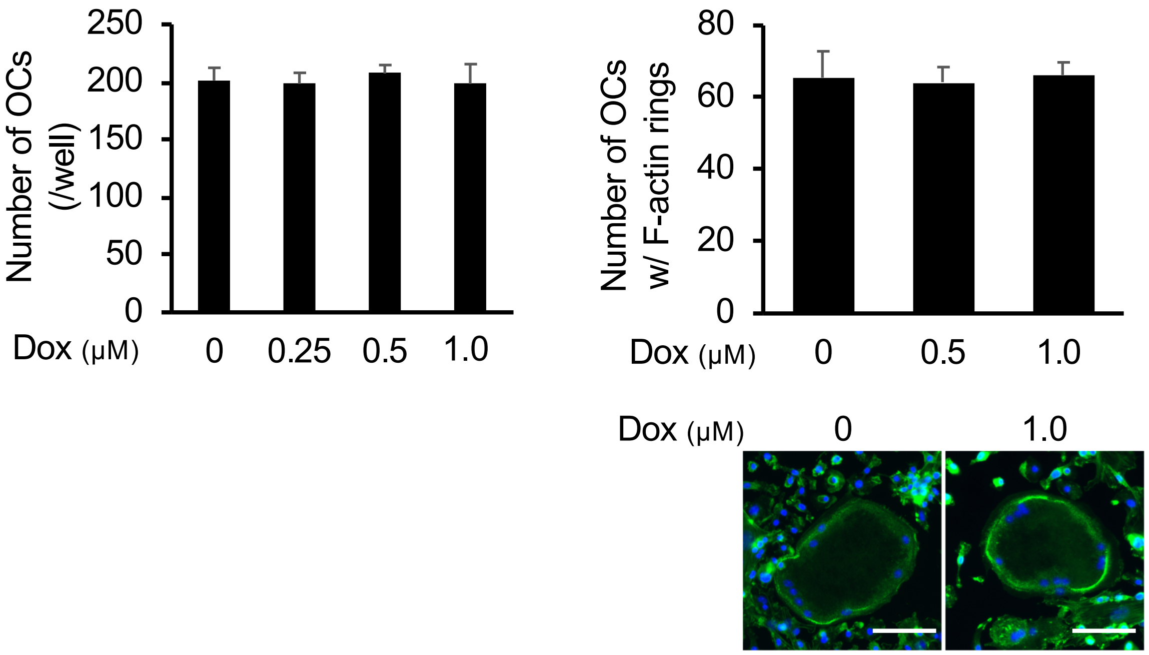

Figure S5. BMMs were cultured with M-CSF (10 ng/mL) and RANKL (50 ng/mL) to generate mature OCs. Then, culture medium was changed and the OCs were cultured in triplicates with the indicated doses of Dox for 48 hours. TRAP-positive multinucleated cells containing three or more nuclei per well were counted (left). Intact OCs were also counted by F-actin ring staining on glass bottom 96-well plates. Representative photos are shown (right). Results are expressed as mean ± SD.

Supplementary Figure 5

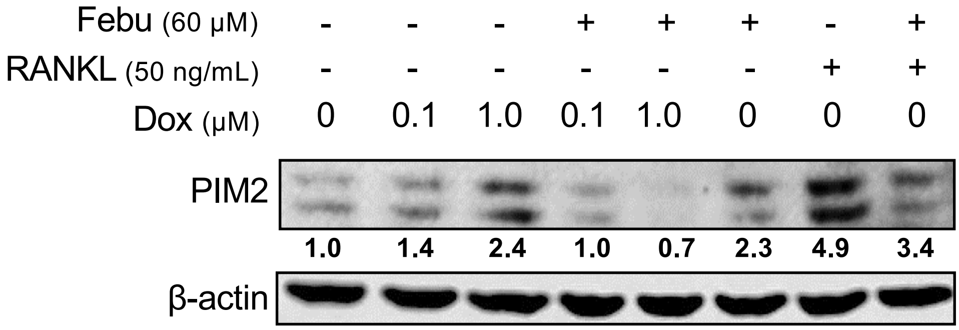

Figure S6. The mature OCs generated from BMMs were cultured with the indicated reagents for 24 hours. Cell lysates were collected, and PIM2 protein levels were analyzed by Western blotting. β -actin served as a loading control. The band sizes of PIM2 was densitometrically compared to those of a control after normalization to those of β-actin.

Supplementary Figure 6

Figure 1E

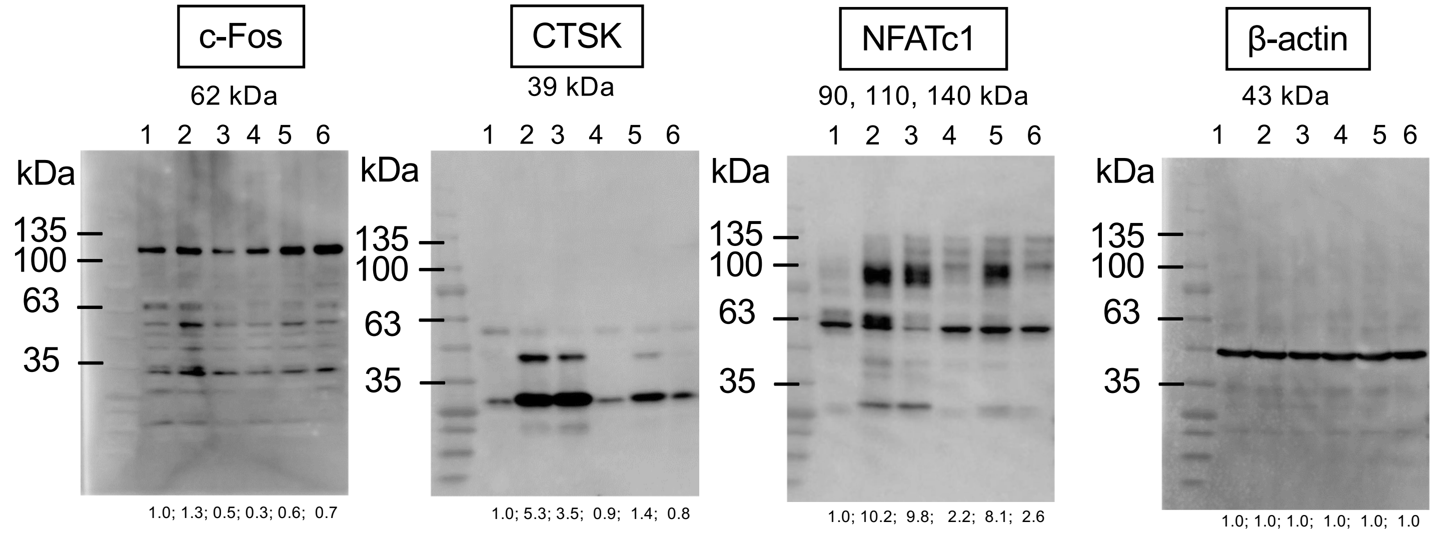

Figure 2C

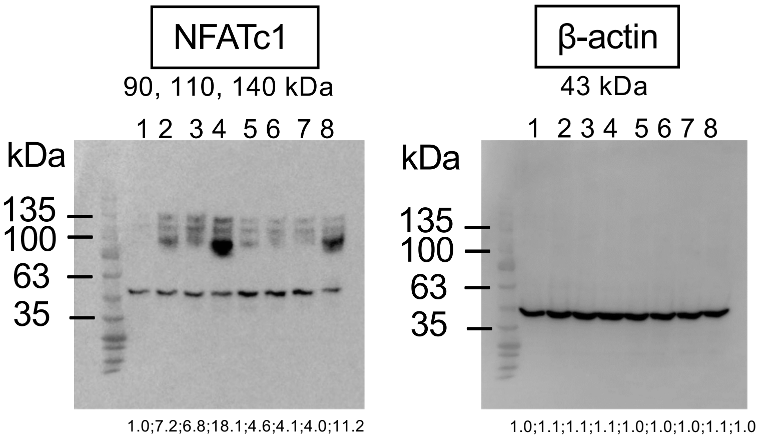

Figure 2D

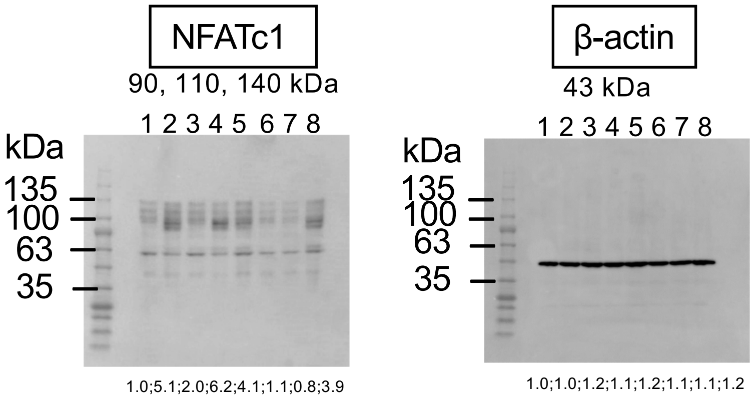

Figure 3B

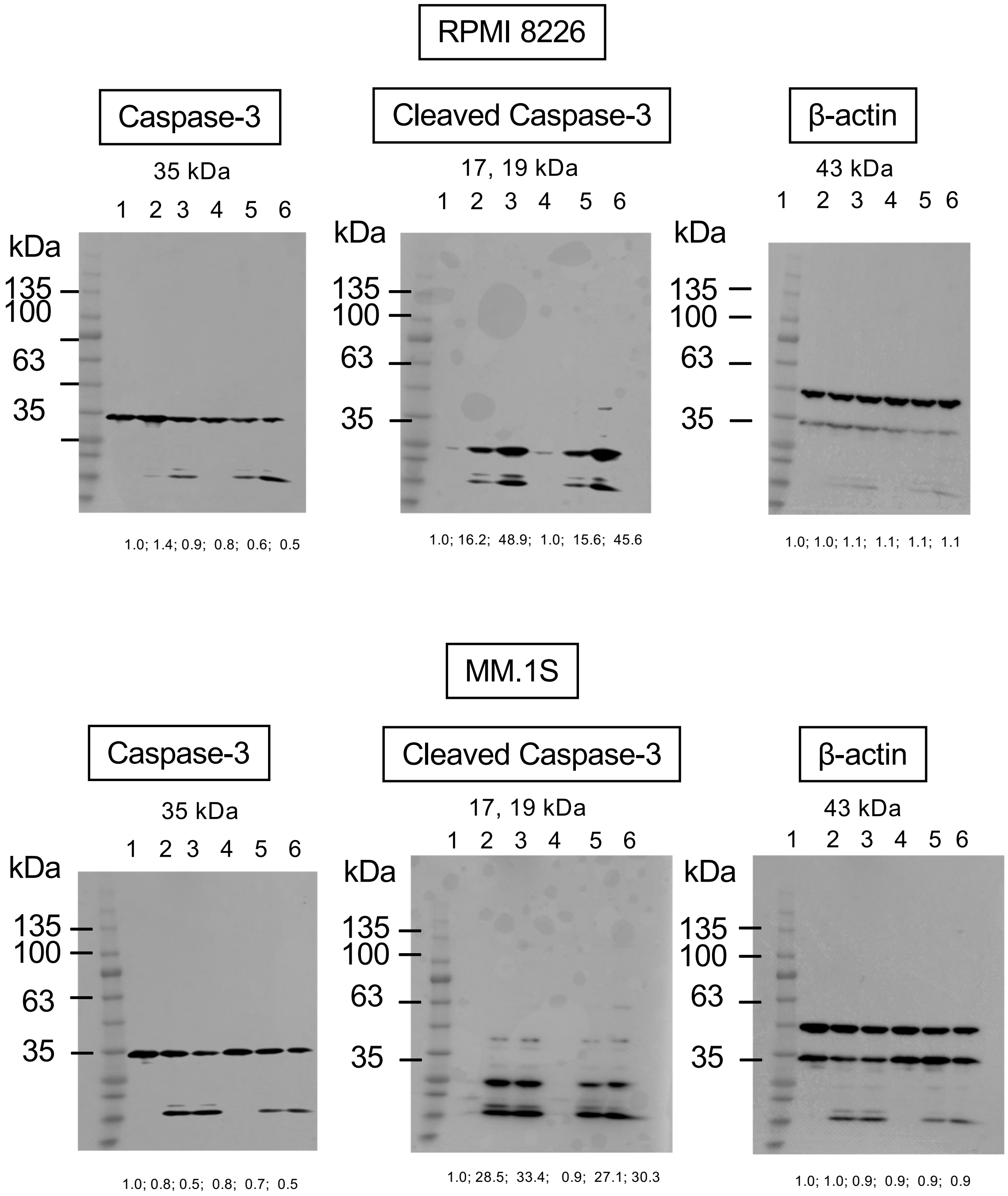
RPMI 8226
Caspase-3
35 kDa
1 2 3 4 5 6
kDa
135
100
63
35
1.0; 1.4; 0.9; 0.8; 0.6; 0.5
Cleaved Caspase-3
17, 19 kDa
1 2 3 4 5 6
kDa
135
100
63
35
1.0; 16.2; 48.9; 1.0; 15.6; 45.6
β-actin
43 kDa
1 2 3 4 5 6
kDa
135
100
63
35
1.0; 1.0; 1.1; 1.1; 1.1; 1.1
MM.1S
Caspase-3
35 kDa
1 2 3 4 5 6
kDa
135
100
63
35
1.0; 0.8; 0.5; 0.8; 0.7; 0.5
Cleaved Caspase-3
17, 19 kDa
1 2 3 4 5 6
kDa
135
100
63
35
1.0; 28.5; 33.4; 0.9; 27.1; 30.3
β-actin
43 kDa
1 2 3 4 5 6
kDa
135
100
63
35
1.0; 1.0; 0.9; 0.9; 0.9; 0.9

Figure S6

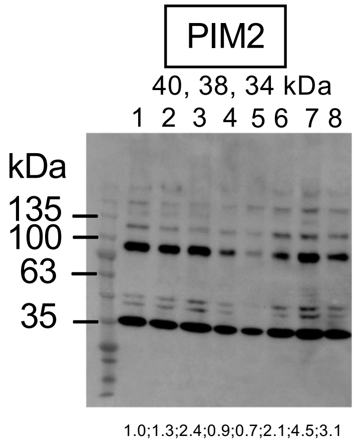

PIM2
40, 38, 34 kDa
1 2 3 4 5 6 7 8
kDa
135
100
63
35
1.0;1.3;2.4;0.9;0.7;2.1;4.5;3.1

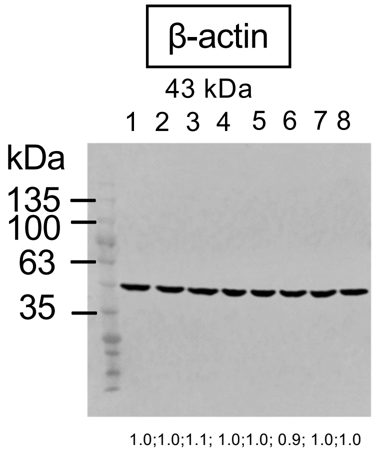

β-actin
43 kDa
1 2 3 4 5 6 7 8
kDa
135
100
63
35
1.0;1.0;1.1; 1.0;1.0; 0.9; 1.0;1.0
